# Data on chemical composition of coffee husks and lignin microparticles as their extracted product

**DOI:** 10.1016/j.dib.2023.109781

**Published:** 2023-11-07

**Authors:** Dung Van Nguyen, Cham Thi Thu Duong, Chau Ngoc Minh Vu, Hung Minh Nguyen, Tuyet Thi Pham, Tuyet-Mai Tran-Thuy, Long Quang Nguyen

**Affiliations:** a Faculty of Chemical Engineering, Ho Chi Minh City University of Technology (HCMUT), 268 Ly Thuong Kiet Street, District 10, Ho Chi Minh City, Vietnam; b Vietnam National University Ho Chi Minh City, Linh Trung Ward, Ho Chi Minh City, Thu Duc City, Vietnam

**Keywords:** Coffee husk, Lignin microparticle, Chemical composition, Biomass valorization

## Abstract

Coffee husks are an abundant and underutilized biomass waste released from coffee production. Experimental analysis showed that coffee husks consisted of 39.2 ± 0.2 wt% cellulose, 12.6 ± 0.1 wt% hemicellulose, 23.3 ± 0.1 wt% Klason lignin, 2.9 ± 0.4 wt% acid-soluble lignin, 8.7 ± 0.2 wt% extractives, and 9.5 ± 0.2 wt% ash. Moreover, different minor elements, including K, Ca, Mg, Al, Fe, Ti, S, and Si, were found. Subsequently, coffee husks were used for the extraction of lignin using an alkaline treatment. As a result, lignin microparticles were formed with a relatively uniform size of 0.55 ± 0.11 mm. Altogether, the current article provided useful data for the valorization of coffee husks and the primary properties of lignin microparticles for further use.

Specifications Table

**Table:** 

Subject	Analytical Chemistry, Materials Chemistry
Specific subject area	Biomass compositional analysis, biomass conversion into useful materials
Data format	Analyzed
Type of data	Tables, Images, Figures
Data collection	Different techniques were used for analyzing the chemical composition of coffee husks. The extractive content was measured through acetone-reflux extraction. A modified Klason technique was applied to evaluate Klason and acid-soluble lignin contents. Holocellulose was extracted via NaClO_2_ treatment. Cellulose and hemicellulose contents were determined by NaOH treatment. The ash content was obtained by incinerating. EDX and FTIR spectra were used to identify minor elements and functional groups, respectively. The morphology of coffee husks was observed by SEM images. Lignin microparticles were extracted from coffee husks using an alkaline treatment. The morphology of lignin microparticles was determined by SEM images. Their elemental composition and functional groups were identified by EDX and FTIR spectra, respectively.
Data source location	Biomass collection: Pleiku City, Gia Lai Province, Vietnam Analysis: Ho Chi Minh City University of Technology (HCMUT), Ho Chi Minh City, Vietnam
Data accessibility	Repository name: Mendeley Data Data identification number: 10.17632/hvr3k6kwf2.2 Direct URL to data: https://data.mendeley.com/datasets/hvr3k6kwf2/2

## 1 Value of the Data

• The chemical composition of coffee husks is important for their further valorization into value-added products.
• The morphology of coffee husk-derived lignin microparticles is useful for future applications.
• Researchers working in the field of biomass valorization and biomass-derived materials can benefit from the data in this article.

## 2 Data Description

Coffee husks are the protective layers of coffee cherries surrounding the coffee beans [1]. The husks are separated from the beans during the harvesting and processing of coffee cherries. Commonly, coffee husks are discarded as waste [2]. In recent years, there has been growing interest in finding sustainable and innovative ways for the use of coffee husks to minimize waste and create value. The biomass can be directly utilized as biomass fuel, organic fertilizer, animal feed, and substrate for mushroom cultivation [3]. Moreover, coffee husks can become a potential raw material for the production of bio-based materials, biofuels, and other value-added products [4,5]. To completely comprehend the features of this resource for future valorization, it is imperative to conduct an analysis of its chemical composition.

**Fig. 1:**
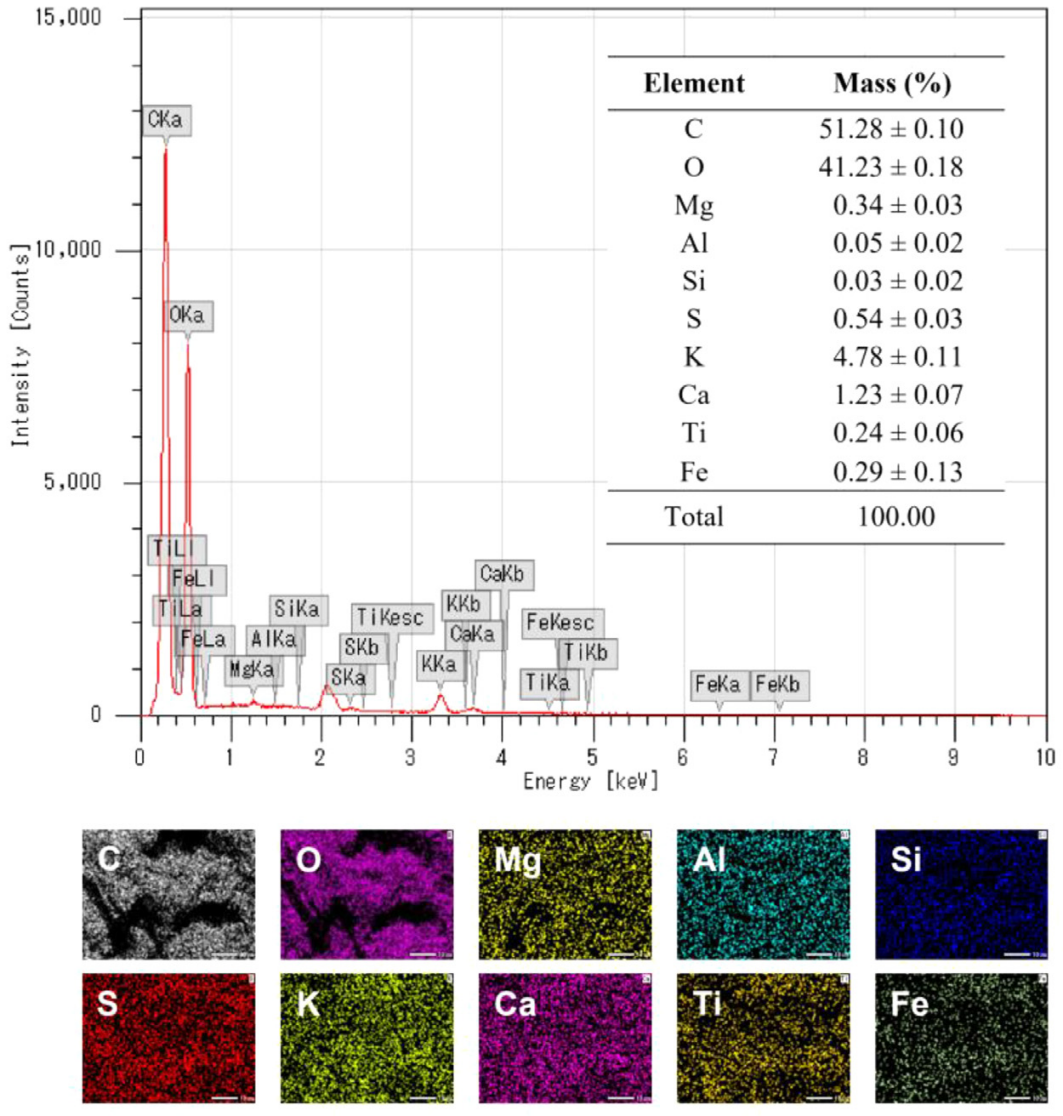
EDX spectroscopy and elemental mapping of coffee husks.

As presented in Table 1, coffee husks contained 39.2 ± 0.2 wt% cellulose, 26.2 ± 0.5 wt% lignin, and 12.6 ± 0.1 wt% hemicellulose. Notably, the ash content in coffee husks reached 9.5 ± 0.2 wt%. Further EDX analysis indicates that minor elements included K, Ca, Mg, Al, Fe, Ti, S, and Si (Fig. 1). Elemental mapping demonstrates that these elements were distributed uniformly throughout the biomass as opposed to being a result of impurities. In addition to acquiring carbon, hydrogen, and oxygen from the surrounding air and water, coffee plants can absorb trace elements from the soil.

**Table 1:** Chemical composition of coffee husks.

Component	Content (wt%)
Cellulose	39.2 ± 0.2
Hemicellulose	12.6 ± 0.1
Klason lignin	23.3 ± 0.1
Acid-soluble lignin	2.9 ± 0.4
Extractives	8.7 ± 0.2
Ash	9.5 ± 0.2
Total	96.2 ± 1.2

**Fig. 2:**
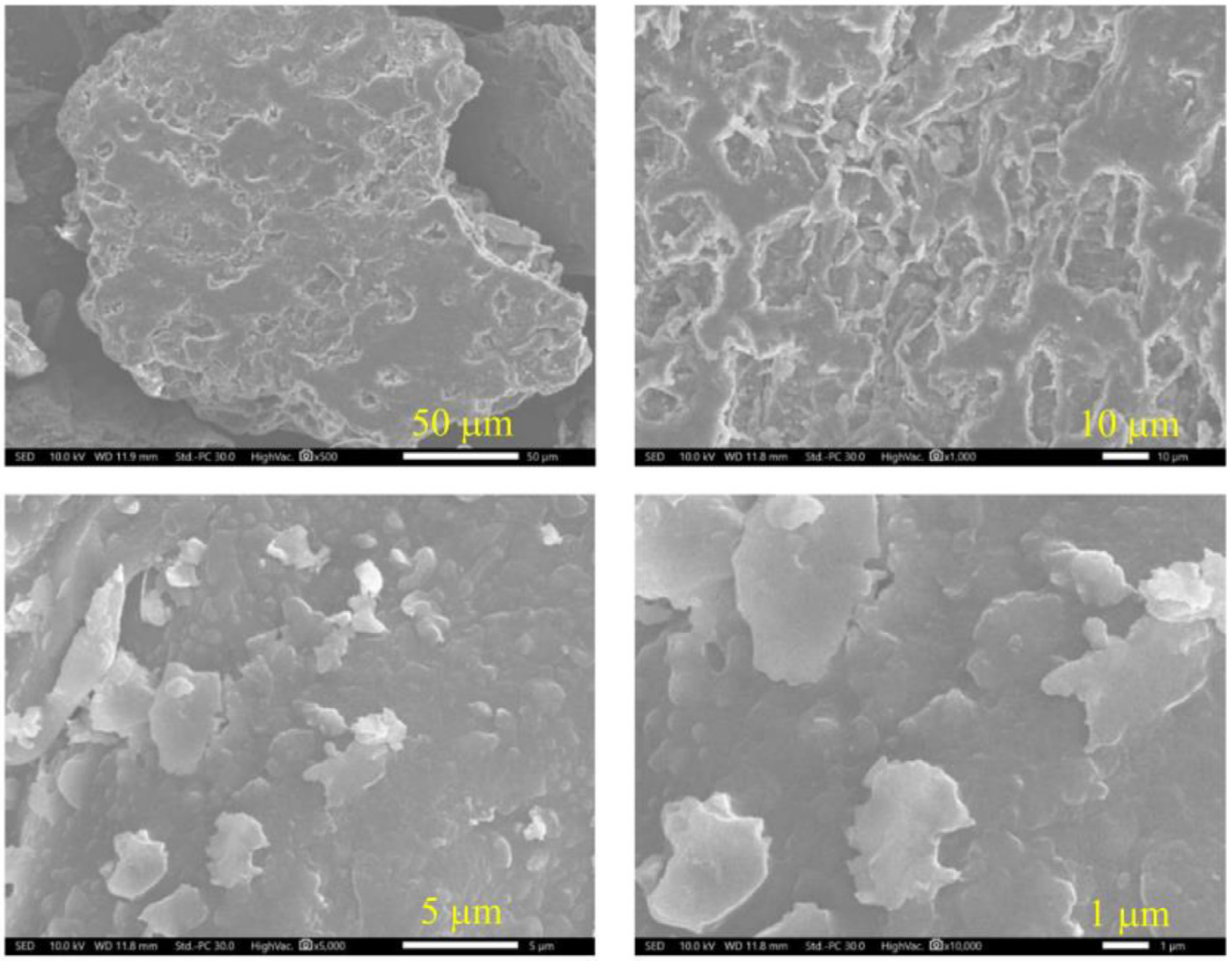
SEM images of coffee husks.

**Fig. 3:**
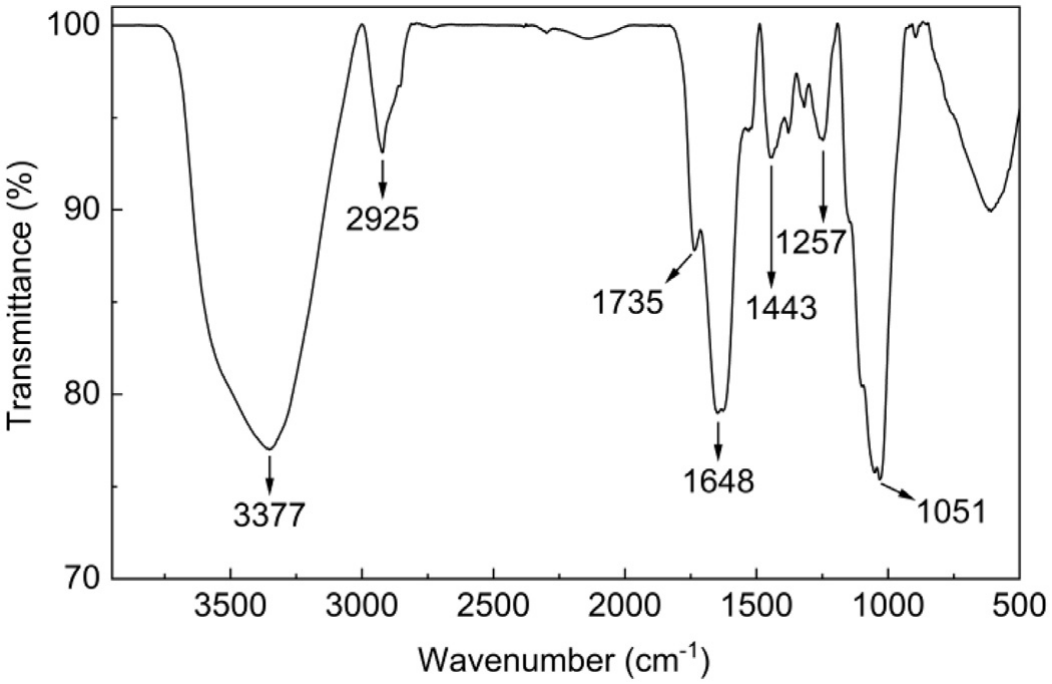
FTIR spectroscopy of coffee husks.

Fig. 2 shows SEM images of coffee husk powder. At the microscale, the biomass had a smooth and flaky morphology. In fact, coffee husks include the outer skin, pulp, and parchment. These layers were ground intensively to offer thin pieces. Moreover, the presence of functional groups in coffee husks was identified by FTIR spectroscopy (Fig. 3). Lignocellulosic biomass contained characteristic peaks: 3377 cm^−1^ (O-H stretching vibrations in cellulose, hemicellulose, and lignin), 2925 cm^−1^ (stretching vibrations of aliphatic C-H bonds in cellulose, hemicellulose, and lignin), 1735 and 1648 cm^−1^ (C=O stretching vibrations in hemicellulose and lignin), 1443 cm^−1^ (C-H bonds in the -OCH_3_ groups of hemicellulose and lignin), 1257 cm^−1^ (stretching vibrations of C-O bonds in lignin), and 1051 cm^−1^ (C-O-C stretching vibrations of cellulose and hemicellulose) [6].

Lignin microparticles have applications in diverse fields, such as materials science, biotechnology, and environmental remediation. When incorporated into polymers, they improve mechanical properties, UV resistance, and thermal stability, promoting the development of sustainable polymer composites through reinforcing or filling functions [7]. These microparticles are also excellent at encapsulating drugs, allowing for targeted delivery, and minimizing side effects [8]. Their biocompatibility expands the possibilities for wound healing and tissue engineering. For agriculture, lignin microparticles serve as slow-release fertilizers and soil enhancers, promoting sustainable plant growth by enhancing soil structure and nutrient accessibility [9]. Due to their porous structure, lignin microparticles are functionalized to efficiently adsorb air and water pollutants [10]. In coatings, films, and packaging, lignin microparticles also enhance barrier properties, UV resistance, color stability, and environmental friendliness [11]. As mentioned before, both cellulose and lignin were main components in coffee husks. However, cellulose extraction from biomass has been reported in great detail in the scientific literature. To broaden the potential uses of coffee husks, lignin extraction was investigated. As a result, coffee husks yielded 13.8 wt% of lignin microparticles. The aforementioned quantity was equivalent to 52.7 wt% of lignin in the raw material.

**Fig. 4:**
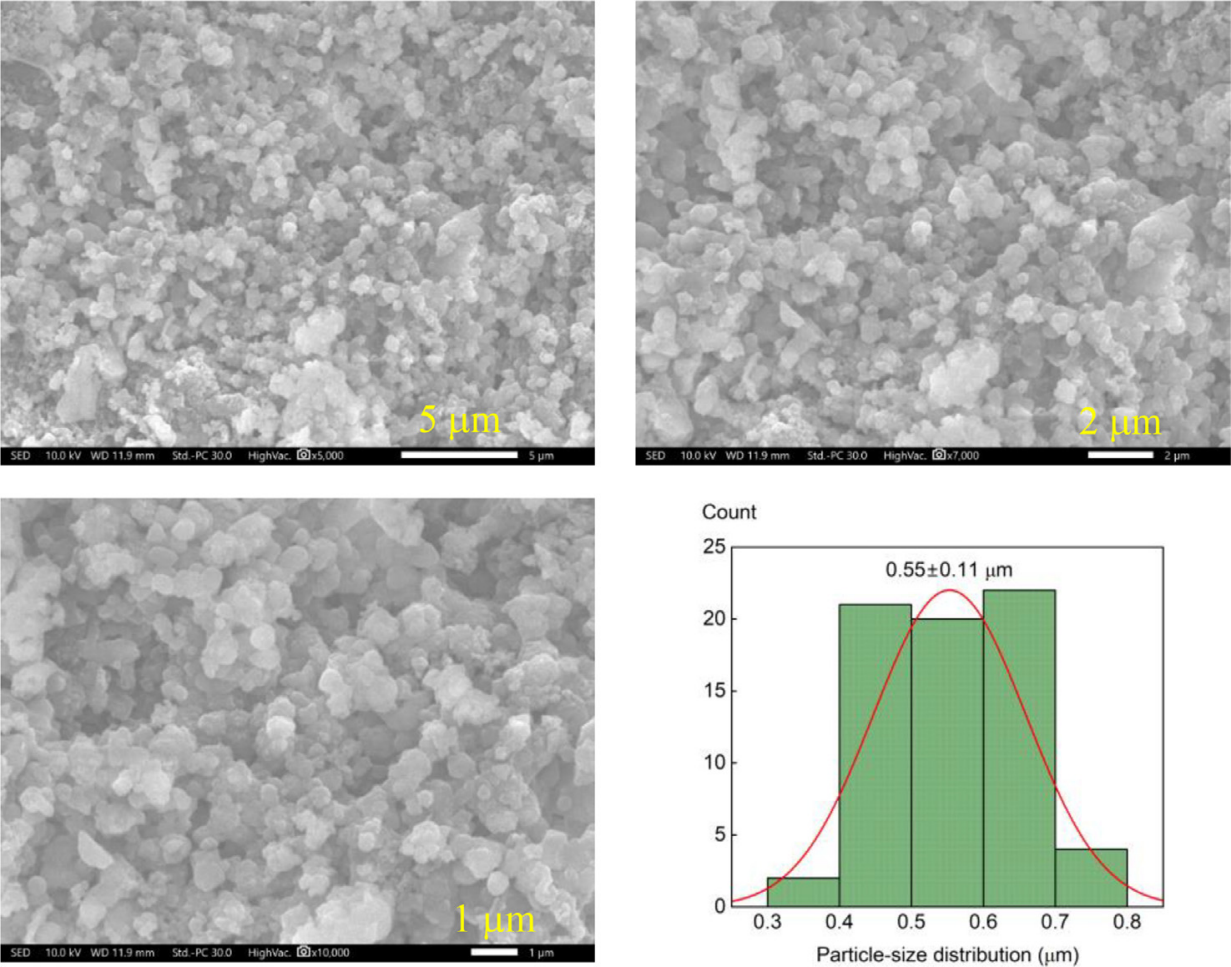
(a) SEM images and (b) size distribution of lignin microparticles.

**Fig. 5:**
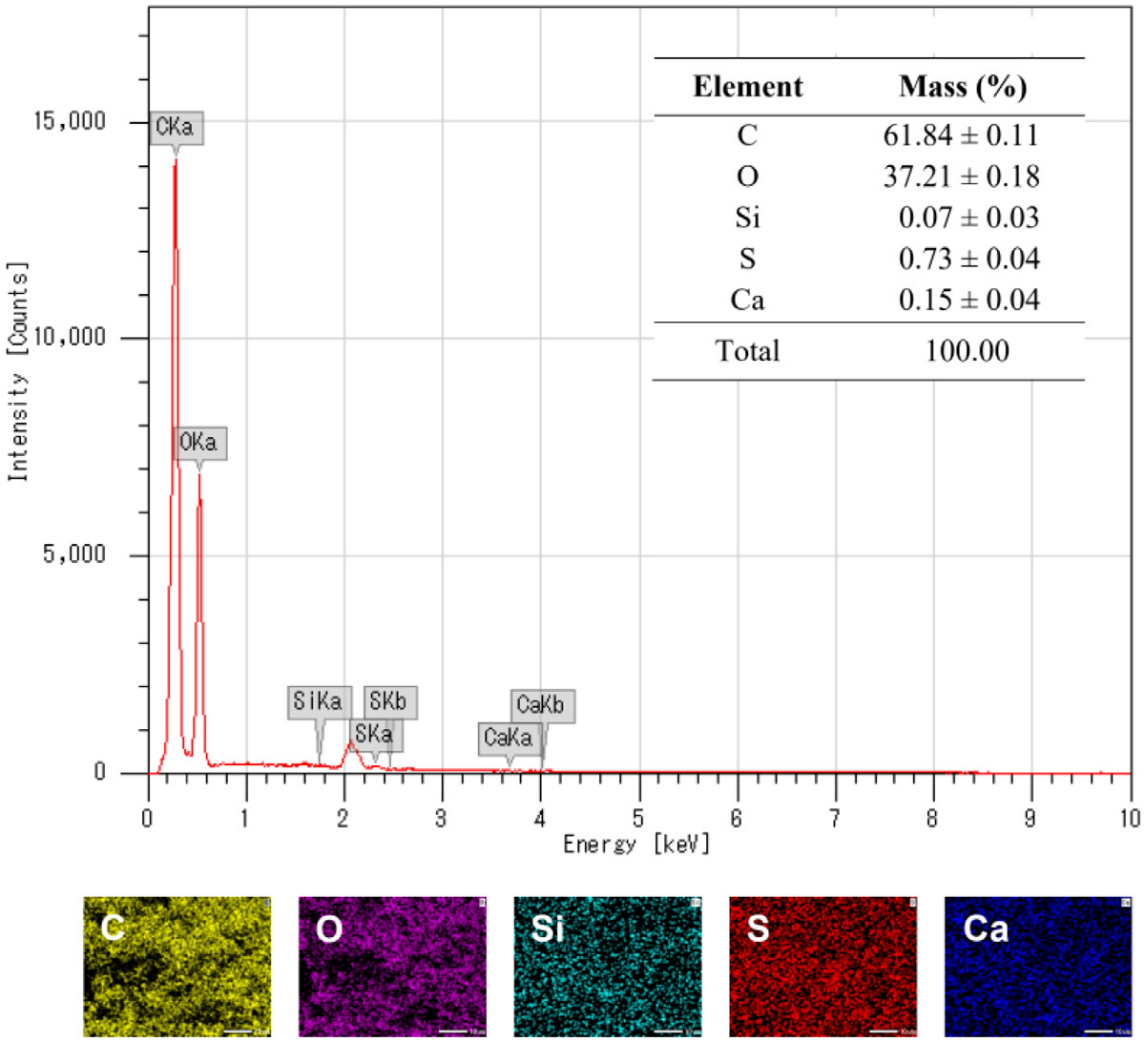
EDX spectroscopy and elemental mapping of lignin microparticles.

SEM images proved that microparticles were formed successfully with an average size and standard deviation of 0.55 ± 0.11 mm (Fig. 4). Compared to coffee husks, lignin microparticles contained more C and less O (Fig. 5). This is because the molar ratio of O to C in lignin is typically lower than that in cellullose and hemicellulose. In addition, the remaining elements S, Si, and Ca exhibited uniform distribution within the lignin structure. Other elements may be lost during the extraction and cleansing processes.

**Fig. 6:**
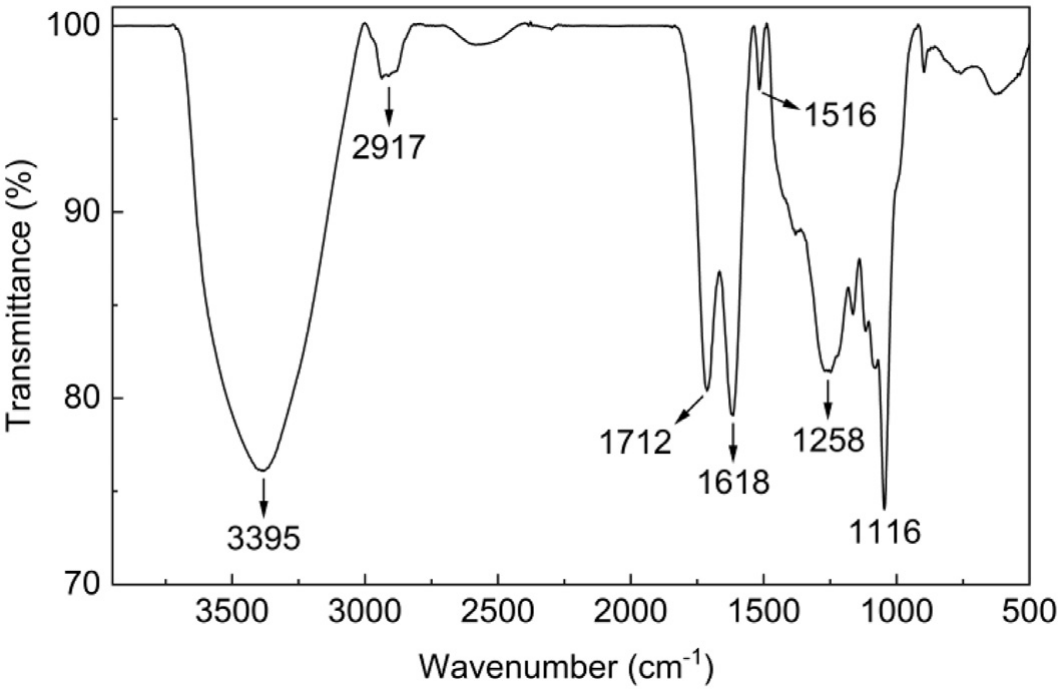
FTIR spectroscopy of lignin microparticles.

As shown in Fig. 6, FTIR spectroscopy of lignin microparticles included characteristic peaks at 3395 cm^−1^ (O-H bonds in phenolic and carboxylic acids), 2917 cm^−1^ (C-H bonds in methyl, methylene, and methoxyl groups), 1712 cm^−1^ (C=O stretching in unconjugated ketones, carbonyl and ester groups), 1618 cm^−1^ (C=O stretching conjugated to the aromatic ring), 1516 cm^−1^ (aromatic ring vibrations), 1258 cm^−1^ (C-O stretching in ether linkages), and 1116 cm^−1^ (C-H bonds in plane deformation) [12], [13], [14]. The successful extraction of lignin microparticles from coffee husks is evidenced by their corresponding functional groups, contrasting with the absence of such groups in cellulose and hemicellulose components.

## 3 Experimental Design, Materials and Methods

### 3.1 Materials

Coffee husks were gathered from the shelling process of coffee beans in Pleiku City, Gia Lai Province, Vietnam. Next, the husks were washed with distilled water, dried at 105 °C for 18 h, and finely ground. This powder was finally put in an airtight plastic bag until it was ready to be used. All chemicals were utilized without further refinement.

### 3.2 Chemical Composition Analysis of Coffee Husks

Each component of coffee husks was determined using a suitable technique. Following is an analysis of the extractive content: 10.0 g of the coffee husk powder was soaked in acetone (60 °C) for reflux extraction [15]. Until the color of the mixture remained unaltered, the extractive-free sample was filtered and dried at ambient temperature.

To quantify Klason and acid-soluble lignin contents, a modified Klason method was implemented [16]. First, 1.00 g of the extractive-free sample was added to 15.0 mL of 72.0 wt% H_2_SO_4_. The mixture was stirred at ambient temperature for 2 h before being diluted to 3.0 wt% H_2_SO_4_. Next, the sample was subjected to autoclaving at 121 °C for 30 min. The solid Klason lignin was obtained by filtering the mixture and drying the residue at 80 °C. The acid-soluble lignin present in the filtrate was analyzed by a Jasco V-730 UV-Vis spectrophotometer equipped with a quartz cuvette. The measurement was conducted at a wavelength of 205 nm, utilizing an absorptivity value of 110 L/g/cm [17].

Holocellulose includes cellulose and hemicellulose. To obtain holocellulose from the extractive-free coffee husk, the NaClO_2_ treatment was applied [18]. First, 2.50 g of the sample was added to 80.0 mL of hot water (80 °C). The suspension was treated with 1.00 g of NaClO_2_ and 0.50 mL of acetic acid at 80 °C for 4 h. This bleaching process was repeated four times. After filtering, rinsing with distilled water, and drying at 80 °C for 24 h, the solid holocellulose was weighted. Next, the holocellulose sample was treated twice with 100 mL of a 17.5 wt% NaOH solution at ambient temperature for 30 min. The solid cellulose was filtered, cleaned with distilled water, desiccated at 80 °C for 24 h, and then weighed. Hemicellulose content was calculated from the difference between holocellulose and cellulose contents. Ash content was determined by incinerating 1.00 g of a dried coffee husk sample at 600 °C for 4 h. All quantitative analyses were performed in duplicate. Average values and standard deviations were calculated by Excel software. Moreover, trace elements present in coffee husks were detected using energy-dispersive X-ray (EDX) spectroscopy, which was collected using the JEOL JSM-IT200 instrument. This instrument was also used to investigate scanning electron microscopy (SEM) images of coffee husks. Before measuring, the dried material was coated with a thin platinum film. The Fourier transform infrared (FTIR) spectroscopy of coffee husks was analyzed using a Bruker Tensor 27 spectrometer.

### 3.3 Extraction of Lignin Microparticles from Coffee Husks

First, 50.00 g of coffee husk powder and 500 g of NaOH solution (5.0 M) were put in a 1 L round-bottom flask. Next, the reflux extraction was conducted at 100 °C for 8 h. Following that, the black solution was collected through vacuum filtration. The solution was precipitated with concentrated H_2_SO_4._ The solid was then washed with distilled water and dried in an oven at 80 °C for 12 h to obtain lignin microparticles (6.91 g). The aforementioned JEOL JSM-IT200 instrument was used to explore SEM images and EDX spectroscopy of the resultant lignin microparticles. Similar to coffee husks, dried lignin microparticles were coated with a thin film of platinum. The size distribution of lignin microparticles was determined by ImageJ and OriginPro software. Lastly, their FTIR spectroscopy was studied by the previously mentioned Bruker Tensor 27 spectrometer.

## Limitations

Not applicable

## Ethics Statement

There are no ethical concerns associated with human subjects, animal experiments, or data collected from social media platforms in this study.

## CRediT authorship contribution statement

**Dung Van Nguyen:** Conceptualization, Writing – review & editing. **Cham Thi Thu Duong:** Investigation. **Chau Ngoc Minh Vu:** Investigation. **Hung Minh Nguyen:** Visualization, Writing – original draft. **Tuyet Thi Pham:** Writing – original draft. **Tuyet-Mai Tran-Thuy:** Methodology. **Long Quang Nguyen:** Methodology.

## Data Availability

Properties of coffee husks and lignin microparticles (Original data) (ICPSR)
